# GaAs-Based InPBi Quantum Dots for High Efficiency Super-Luminescence Diodes

**DOI:** 10.3390/ijms20236001

**Published:** 2019-11-28

**Authors:** Liyao Zhang, Yuxin Song, Qian Gong

**Affiliations:** 1 Department of Physics, University of Shanghai for Science and Technology, Shanghai 200093, China; 2 State Key Laboratory of Functional Materials for Informatics, Shanghai Institute of Microsystem and Information Technology, Shanghai 200050, China; songyuxin@gmail.com

**Keywords:** InPBi, quantum dot, finite element method, super-luminescent diode

## Abstract

InPBi exhibits broad and strong photoluminescence at room temperature, and is a potential candidate for fabricating super-luminescence diodes applied in optical coherence tomography. In this paper, the strained InPBi quantum dot (QD) embedded in the AlGaAs barrier on a GaAs platform is proposed to enhance the light emission efficiency and further broaden the photoluminescence spectrum. The finite element method is used to calculate the strain distribution, band alignment and confined levels of InPBi QDs. The carrier recombinations between the ground states and the deep levels are systematically investigated. A high Bi content and a flat QD shape are found preferable for fabricating super-luminescence diodes with high efficiency and a broad emission spectrum.

## 1. Introduction

Dilute bismides are a group of compound semiconductors formed by incorporating a small amount of Bi atoms into the traditional III-V semiconductors. They show a lot of special properties, such as large bandgap bowing, spin-orbit splitting energy increment and temperature insensitivity of the bandgap [1,2,3]. InPBi is a member of the dilute bismides, first realized by molecular beam epitaxy (MBE) in 2013 [4]. It exhibits the strongest bandgap reduction of 106 meV/Bi% [5], compared with other dilute bismides. The energy shifts of the conduction band (CB) and valence band (VB) for InPBi are −27 meV/Bi% and 79 meV/Bi%. Furthermore, InPBi thin film has a unique property in that it possesses a broad and strong photoluminescence (PL) spectrum at room temperature, involving not only the band edges but also the deep levels. The wide PL spectrum is formulated by the carrier recombinations from the CB to a Bi-related acceptor level, from a P_In_ antisite donor level to the VB and between the two deep levels [6]. Optical coherence tomography (OCT) is a non-invasive medical imaging technique, in which the axial resolution is directly related to the spectrum width of the light source [7], i.e., the wider the spectrum, the better the axial resolution. The broad and strong PL of InPBi thin film at room temperature makes it a potential material for fabricating super-luminescence diodes (SLDs) applied in OCT [8]. Recently, the InPBi quantum dot (QD)/InAlAs on InP was predicted to be an effective structure to provide confinement for the carriers to increase photon emission efficiency and further broaden the PL spectra [9]. However, when investigating the barrier material for the InPBi QD on the InP lattice-matched systems, such as InAlAs, InGaAs and AlAsSb, the Bi content should be over 2%, 7.4% and 6.1%, respectively, to ensure a type-I band alignment. Bi incorporation is challenging in dilute bismides [10,11,12,13], and the reported highest Bi content incorporated in InPBi thin film is 3.4% [5]. Even if a type-I potential well is formed, the well depth is shallow, resulting in poor carrier confinement. The lack of lattice mismatch between the InPBi and the InP makes it impossible to be experimentally realized through the common Stranski–Krastanov (S–K) growth mode for III-V QDs.

In this work, we propose a new structure of InPBi QD embedded in AlGaAs on a GaAs platform. First, the AlGaAs/InP structure can easily form a type-I band alignment when the Al content is over 41.7%, making it straightforward for an InPBi QD/AlGaAs structure to also possess a type-I band alignment with a shrinking bandgap. Second, the lattice constant of GaAs is much smaller than that of the InP and the InPBi, leading to substantial compressive strain platforming the InPBi QDs, which can not only trigger the S–K growth mode but also shift the band edges [14] to further broaden the PL spectra. The finite element method (FEM) is used to calculate the strain distribution, as well as the electronic band structures, including band edges and ground states of electrons, heavy and light holes. The hydrostatic strain and shear strain components are first simulated with different Bi content and morphology of the InPBi QD. Then, the band alignment is calculated based on deformation potential theory [15]. Through controlling the Bi content and morphology of the InPBi QDs, the carrier recombination energy can be tailored. The InPBi QD/AlGaAs on GaAs structure proposed in this work provides a realizable way based on InPBi to fabricate SLDs applied in OCT.

## 2. Results and Discussions

### 2.1. Strain Analysis

**Figure 1:**
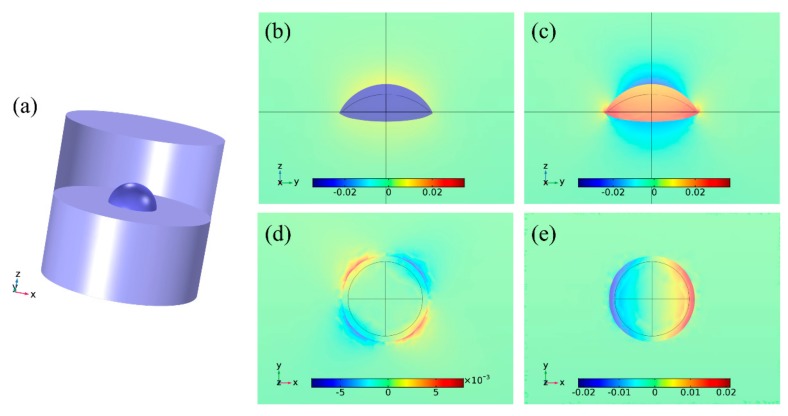
(**a**) Three-dimensional schematic of the proposed InPBi QD/AlGaAs structure. The strain distribution of (**b**) *ε_xx_* and (**c**) *ε_zz_* in the *yz* plane; and (**d**) *ε_xy_* and (**e**) *ε_xz_* in the *xy* plane for the InPBi QDs in Al_0.42_Ga_0.58_As with a Bi content of 2%, a diameter of 20 nm and a height of 4 nm. The deformations in (**b**,**c**) and (**d**,**e**) are exaggerated by 20 times and 50 times, respectively.

The strain distributions are calculated with the model shown in Figure 1a. An InPBi QD in a spherical cap shape is buried in AlGaAs. A representative model with the Bi content, diameter and height of 2%, 20 nm and 4 nm, respectively, is firstly simulated. Figure 1b–d show the strain components of *ε_xx_* (b) and *ε_zz_* (c) in the *yz* plane across the center of the QD; and *ε_xy_* (d) and *ε_xz_* (e) in the *xy* plane across the bottom of the QD. The *ε_xx_* in the QD is negative and uniform with an average value of −0.03, indicating a compressive in-plane strain. This is due to the larger lattice constant of InPBi, comparing to that of AlGaAs. The *ε_xx_* in AlGaAs is positive and rapidly decreases from the AlGaAs/InPBi QD interface. The *ε_zz_* in the QD is positive with an average value of 0.02, indicating a tensile strain in the *z* direction. The *ε_zz_* in the QD is non-uniform with a larger value at the bottom. The *ε_zz_* in AlGaAs is negative above and below the QD and decreases to zero away. The shear strain components *ε_xy_* (d) and *ε_xz_* (e) are asymmetric in the *xy* plane and their average value are 1.0E-6 and 4.8E-6, respectively. The average value in the QD of *ε_xx_* and *ε_zz_* are four orders of magnitude larger than and roughly one order larger than that of *ε_xy_* and *ε_xz_*.

**Figure 2:**
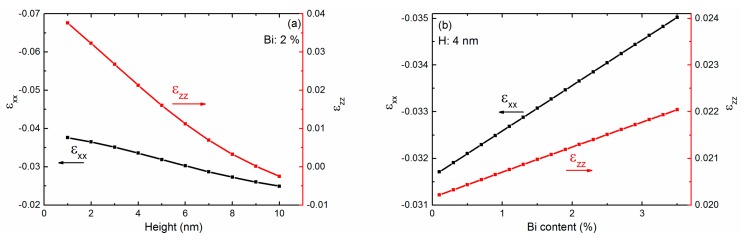
The variation of the strain components of *ε_xx_* and *ε_zz_* in the *yz* plane with the height (**a**) and Bi content (**b**) of the InPBi QD. The Bi content and the diameter are set at 2% and 20 nm, respectively in (**a**), while the diameter and height are set at 20 nm and 4 nm, respectively in (**b**).

The influence of the Bi content and size of the QD on the strain is investigated systematically. The variations of *ε_xx_* and *ε_zz_* with the height (a) and Bi content (b) are shown in Figure 2. The left and right longitudinal axes represent *ε_xx_* and *ε_zz_*, respectively. The Bi content and diameter are set at 2% and 20 nm, respectively, in Figure 2a. *ε_xx_* is negative when the height varies from 1 to 10 nm. The absolute value of *ε_xx_* decreases with an increase in the height of the QD. *ε_zz_* is positive and decreases with the height of the QD varying from 1 to 9 nm. When the height increases to 10 nm, the QD becomes a hemispherical shape, and *ε_zz_* becomes negative. The absolute value of *ε_xx_* is larger than that of *ε_zz_*. The difference between the absolute value of *ε_xx_* and *ε_zz_* increases as the height increases. The absolute value of *ε_xx_* and *ε_zz_* both decrease monotonously with the height, with slopes of about −0.0015/Bi% and −0.0046/Bi%, respectively. In Figure 2b, the diameter and height of the QD are set at 20 nm and 4 nm, respectively. *ε_xx_* is negative while *ε_zz_* is positive. The absolute value of *ε_xx_* and *ε_zz_* both increase linearly with the Bi content, with slopes of 0.001/Bi% and 0.0005/Bi%, respectively.

**Figure 3:**
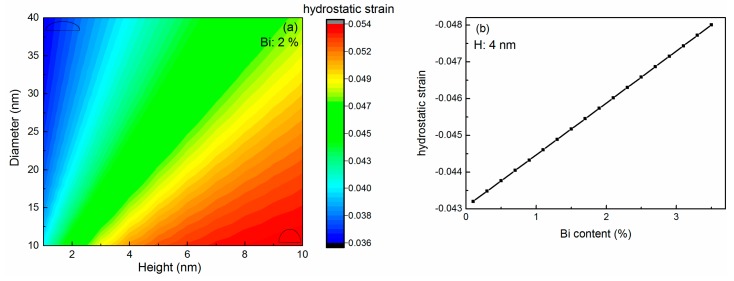
The hydrostatic strain with different aspect ratios (**a**) and Bi contents (**b**). The Bi content is set at 2% in (**a**). The two spherical cap shapes at the top left and bottom right corner show the corresponding shapes of the QD. The diameter and height are set at 20 nm and 4 nm, respectively, in (**b**).

The hydrostatic strain, which is the summary of *ε_xx_*, *ε_yy_* and *ε_zz_*, is further discussed with the shape and Bi content of the InPBi QD. The Bi content is set at 2% in Figure 3a. The diameter and height vary from 10 to 40 nm and 1 to 10 nm, respectively. The two spherical cap shapes at the top left corner and bottom right corner represent the corresponding shapes of the QD with the aspect ratio of diameter to height of 4:1 and 1:1, respectively. The hydrostatic strain is negative in the variation ranges of the diameter and the height. It is the same for the QDs of the same aspect ratio (D/H) and increases when the D/H decreases. This indicates that a flatter InPBi QD buried in AlGaAs holds a smaller compressive hydrostatic strain. In Figure 3b, the diameter and height are set at 20 nm and 4 nm, and the Bi content varies from 0.1% to 3.5%. The hydrostatic strain is also negative and linear to the Bi content, with a slope of −0.0014/Bi%. Bi incorporation can introduce larger compressive hydrostatic strain into the QD.

### 2.2. Band Structure

**Figure 4:**
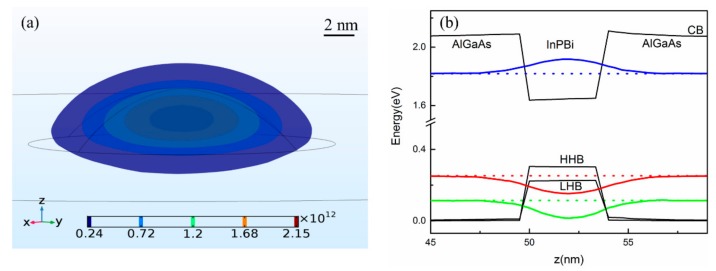
(**a**) 3D distribution of electron wave functions for the ground state, plotting in isosurfaces. (**b**) band alignment and distribution of wave functions for the ground states of electrons, heavy holes and light holes. The black solid lines represent the CB, heavy holes band (HHB) and light holes band (LHB), respectively. The blue, red and green curves and dotted ones represent the real part and the imaginary part of the wave functions, respectively.

The band alignment and wave functions are calculated for the InPBi QD/AlGaAs structure, with one example shown in Figure 4. The Bi content, diameter and height for the calculated structure are 2%, 20 nm and 4 nm, respectively. 

Figure 4a shows the 3D distribution of the electron wave function for the ground state plotted in isosurfaces. The wave function concentrates in the center of the QD and decreases for more than one order of magnitude around the interface of the InPBi QD and AlGaAs. A similar situation is found for the holes. Figure 4b shows the band alignment and the distribution of the wave function for the ground states of electrons and holes cross the QD center in the height direction. The heavy hole band (HHB) is above the light hole band (LHB), which is due to the compressive strain in the InPBi QD. It can be observed that the wavefunctions of the electrons and the holes are mostly confined in the QD and well overlapped spatially. The effective 3D confinement and the wavefunction overlap for the electrons and holes are key factors to enhance the carrier recombination rate and subsequently the light emission efficiency of LED.

**Figure 5:**
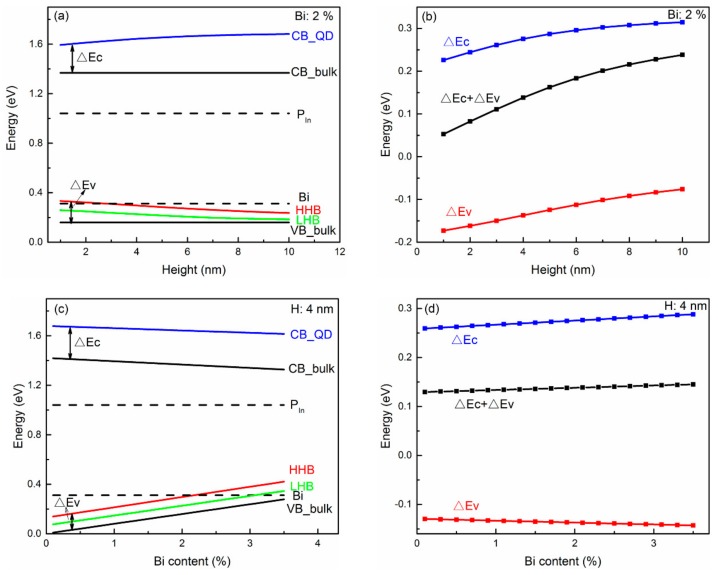
Diagram of the CB and VB of the InPBi QD with the height (**a**) and Bi content (**c**), comparing to InPBi bulk material. The differences of the CB and VB between the InPBi QD and bulk with the height (**b**) and Bi content (**d**). The Bi content and height of the QD are set at 2% and 4 nm, respectively in (**a**,**b**) and (**c**,**d**). In (**a**,**c**), the blue, red and green lines are the CB, HHB and LHB of InPBi QD, respectively. The black lines are the CB and VB of InPBi bulk material, respectively. The dashed lines are the P_In_ antisite and Bi-related deep levels, respectively.

**Figure 6:**
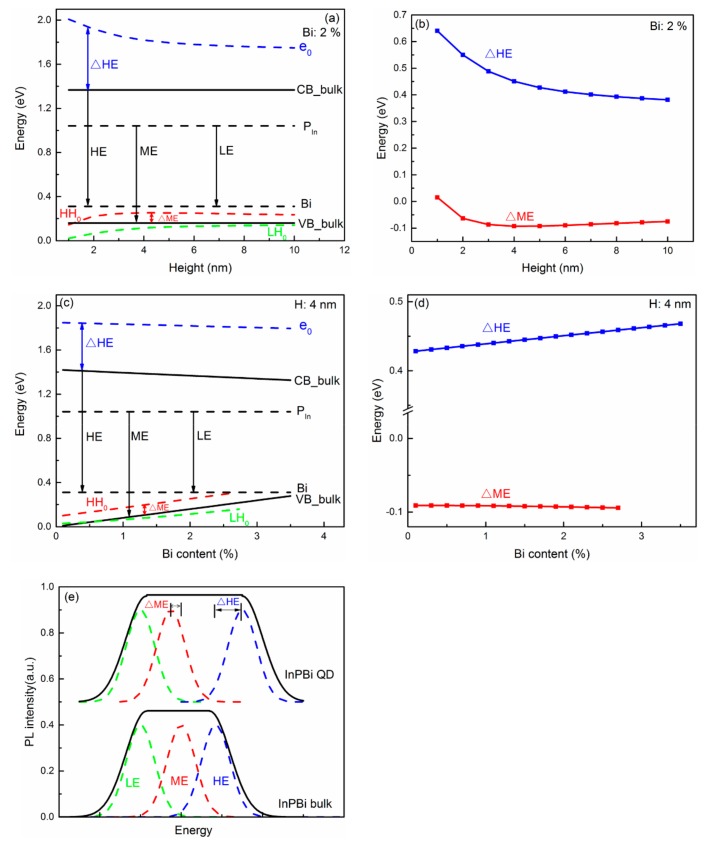
Diagram of carrier recombination of InPBi QD/AlGaAs structure plotted along the *z* axis across the center of the InPBi QD with the Bi content of 2% (**a**,**b**) and the height of 4 nm (**c**,**d**), respectively. (**e**) sketch of the broadening of PL spectra of InPBi QD vs. InPBi bulk. The blue, red and green dashed curves in (**a**,**c**) are the ground states of electrons, heavy holes and light holes, respectively. The blue and red curves in (**b**,**d**) are the energy difference between the ground states of electrons of InPBi QD and the CB of InPBi bulk, and the VB of InPBi bulk and the ground states of heavy holes of InPBi QD, respectively. The black, green, red and blue curves represent the PL, LE transition, ME transition and HE transition of the InPBi.

The band structure of the InPBi QD and bulk material is compared and summarized in Figure 5 and Figure 6. The zero energy is set at the top of the VB of AlGaAs. The diameter of the InPBi QD is set at 20 nm in the following discussions.

Figure 5a,c show the diagram of the band structure of InPBi QD with different heights (a) and Bi contents (c). The bottom of the CB of the InPBi QD is above that of the InPBi bulk material, while the top of HHB and LHB of the InPBi QD is above the VB of the bulk material, showing that the compressive strain in the QD increases the band edge energy in the conduction band while decreasing that of the VB, unlike the common case of InGaAs on GaAs. Usually, compressive strain induces the rise in the CB and the decline of the VB, resulting in a bandgap enlargement. However, different phenomena are also observed in [16]. Kwiseon Kim, et al., calculated the band edge of InAs/GaAs and InAs/InP. They found that the compressive strain in InAs increases the CB, HHB and LHB of InAs, which is similar to the InPBi case. The difference between the CB of the InPBi QD and the bulk material is labeled as ΔEc. The difference between the VB of the InPBi bulk material and the HHB of the InPBi QD is labeled as ΔEv. Thus, the bandgap increment is ΔEc + ΔEv. Figure 5b,d show the variation of ΔEc, ΔEv and ΔEc + ΔEv with the QD height and the Bi content, respectively. As shown in Figure 5b, ΔEc is positive and increases as the height increases. The absolute value of the hydrostatic strain increases as the height increases, as discussed above. So, the strain-induced CB shift is linear to the hydrostatic strain with the slope of *a_c_*, as in formula (3). ΔEv is negative and increases as the height increases. ΔEv is related to two items, shown in expressions (4), (6) and (7). The hydrostatic-strain-related energy increases with the height, while the (*ε_xx_ + ε_yy_ −* 2*ε_zz_*)-related energy decreases with the height. The overall strain-induced energy shift for the VB increases with the QD height. The bandgap increment from ΔEc to ΔEv is positive and increases with the height. The compressive strain increases the CB, HHB and LHB, but the lift of the CB exceeds the lift of the VB, resulting in a bandgap increment. In Figure 5d, ΔEc is positive and increases as the Bi content increases. This is due to the increase in the absolute value of the hydrostatic strain with increasing Bi content. ΔEv is negative and decreases with increasing Bi content. The hydrostatic-strain-related energy increment is smaller than the (*ε_xx_* + *ε_yy_* − 2*ε_zz_*)-related energy decrease. The bandgap of the InPBi QD is enlarged under compressive strain. The bandgap enlargement increases with Bi content. 

Figure 6 shows the carrier recombination processes in the InPBi QD and bulk material. The ground state of electrons (e_0_), heavy holes (HH_0_) and light holes (LH_0_) is calculated with different height (a) and Bi content (c). The black lines are the CB and VB of InPBi bulk, respectively. The dashed lines are the P_In_ antisite deep level and Bi-related deep level, respectively. The three major carrier recombination processes are from the CB_bulk to the Bi-related deep level, from the P_In_ antisite deep level to the VB_bulk, and from the P_In_ antisite deep level to the Bi-related deep level, which are labeled as HE, ME and LE, respectively. The energy shifts for the HE and ME are labeled as ΔHE and ΔME, respectively. In Figure 6a,b, the Bi content is set at 2%, and the height varies from 1 to 10 nm. e_0_ is above the CB_bulk and LH_0_ is below the VB_bulk. HH_0_ is below the VB_bulk when the height is 1 nm and above the VB_bulk when the height exceeds 1 nm. e_0_ decreases with height, while HH_0_ and LH_0_ increase with height. This is due to the abatement of the quantum confinement effect with an increasing height. ΔHE is positive and decreases with height, as shown in Figure 6b. The CB of the InPBi QD rises with height, which increases the ΔHE. However, the quantum confinement effect is weakened with increasing height, causing a smaller e_0_, and a decreased ΔHE. The total effects result in a decrease in ΔHE with increasing height. ΔME is positive when the height is 1 nm and turns negative when the height is above 1 nm. ΔME decreases when the height varies from 1 to 4 nm and then increases with height. The HHB descends with increasing height, thus increasing the ΔME. Meanwhile, decreasing the HHB of the InPBi QD would decrease the energy difference between AlGaAs and InPBi, weakening the quantum confinement effect. This would increase the energy of HH_0_, thus decreasing ΔME. It is probable that the quantum confinement effect for holes is dominant when the height is below 4 nm while the descending HHB for holes is dominant when the height is over 4 nm. 

Figure 6c,d show the energy variations following different Bi contents with a fixed QD height of 4 nm. e_0_ linearly decreases with Bi content, with a slope of −0.015 eV/Bi%. HH_0_ and LH_0_ linearly increase with Bi content, with slopes of 0.08 eV/Bi% and 0.05 eV/Bi%, respectively. HH_0_ overlaps with the Bi-related deep level when the Bi content is over 2.7%, and the situation is complicated. So, we only discuss the situations when the Bi content is below 2.7%. ΔHE is positive and linearly increases with the Bi content, with a slope of 0.01 eV/Bi%. The CB of the InPBi QD decreases with an increasing Bi content, which decreases ΔHE. However, decreasing the CB enlarges the energy difference between InPBi and AlGaAs, enhancing the quantum confinement. The energy of e_0_ would increase, thus increasing ΔHE. The Bi-content-induced quantum confinement is dominant over the CB reduction. ΔME is negative and almost unchanged with the Bi content. The effect on the holes of the rise in the HHB is almost cancelled out by the weakening of the quantum confinement.

Figure 6e shows the schematic of the broadening of the PL spectrum of the InPBi QD compared with InPBi bulk. Mostly due to the right shift of HE, the PL spectrum could be broadened. 

The ultimate purpose of proposing the InPBi QD/AlGaAs structure is to fabricate SLDs with broad and bright spectra. A flat InPBi QD with a high Bi content (*x* ≤ 2.7%) would be the optimal choice.

The InPBi QDs can be realized in a S–K growth mode, since the lattice mismatch between InPBi and AlGaAs is large enough. Liquid droplet epitaxy can also be employed. However, the liquid phase crystallization of InPBi would be quite different from the common MBE process, and the optical property would subsequently be different. The structural properties of InPBi QDs could be studied through atomic force microscopy and transmission electron microscopy, while the optical properties could be analyzed though photoluminescence. 

## 3. Methods

The proposed InPBi QD/AlGaAs structure is schematically shown in Figure 1a. The InPBi QD was assumed to be in a spherical cap shape, with the Bi content, diameter and height varying in the range of 0.1–3.5%, 10–40 nm and 1–10 nm, respectively. The InPBi QD was buried in AlGaAs. The Al content was set at 42% in this work, slightly over 41.7% which ensured a type-I band alignment. FEM was applied to calculate the strain distribution and the band structure in the proposed structure. The lattice constants of InP (*a_InP_*), InBi (*a_InBi_*), AlAs (*a_AlAs_*) and GaAs (*a_GaAs_*) were 5.87 Å, 6.52 Å, 5.66 Å and 5.65 Å [4,17], respectively. The lattice constants of InP_1-*x*_Bi*_x_* (*a_InPBi_*) and Al_y_Ga_1-y_As (*a_AlGaAs_*) were deduced to be (1 − *x*)*a*_InP_ + *xa*_InBi_ and (1 − *y*)*a_GaAs_* + *ya_AlAs_* based on the Vegard’s law assumption. The lattice mismatch between InPBi and AlGaAs was defined as (*a_InPBi_* − *a_AlGaAs_*)/*a_InPBi_*. The elastic coefficients C11, C12 and C44 of InPBi and AlGaAs were also deduced from a linear interpolation between InP and InBi, and AlAs and GaAs, respectively. With the computed strain distribution as an input, the band structure was calculated based on deformation potential theory. Considering the strain effect, the Schrödinger equation is

[−*ħ*^2^/(2m*)∇^2^ + *V*]*ψ*(*r*) = *Eψ*(*r*) (1)

*V* = *V*_0_ + *V_s_* (2)

where *V*_0_ is the band offset between InPBi and AlGaAs, *V_s_* is the strain-induced potential, *ħ* is the reduced Planck constant and m* is the effective mass of the carriers. We calculated an *8*-band strain-dependent *k·p* Hamiltonian. The 8 × 8 Hamiltonian matrix is *H*_0_ + *H_s_*, where *H*_0_ is the kinetic component of Hamiltonian and *H_s_* is the strain-related Hamiltonian.

*a_c_ε_hy_*0−v*0−3^½^*v*2^½^*u**u*−2^½^v*    (3)
0*a_c_ε_hy_*2^½^*u*3^½^v*0*v*−2^½^*v*−*u*
−*v*2^½^*u*−*P* + *Q*−s**r*01.5^½^*s*−2^½^*q*
03^½^*v*-*s*−*P* − *Q*0*r*−2^½^*r*s/2^½^*H*_*s*_ =−3^½^v*0r*0−*P* − *Q*s*s*/2^½^2^½^r*
2^½^*u*v*0r**s*−*P* + *Q*2^½^*q*1.5^½^s*
*u*−2^½^v*1.5^½^s*−2^½^r**s*/2^½^2^½^*q*-*a_v_ε_hy_*0
−2^½^*v*−*u*−2^½^*q*s*/2^½^2^½^*r*1.5^½^*s*0−*a_v_ε_hy_*
where

*ε_hy_* = (*ε_xx_* + *ε_yy_* + *ε_zz_*) (4)

*P* = −*a_v_* (*ε_xx_* + *ε_yy_* + *ε_zz_*) (5)

*Q* = −*b* (*ε_xx_* + *ε_yy_* − 2*ε_zz_*)/2 (6)

*r* = 0.75^½^*b* (*ε_xx_* − ε_yy_) − *idε_xy_* (7)

*s* = −*d* (*ε_xz_* − *iε_yz_*) (8)

*u* = −*iP_0_*∑*ε_zj_∂_j_*/3^½^ (9)

*v* = −*iP_0_*∑(*ε_xj_* − *iε_yj_*)*∂_j_*/6^½^ (10)

*ε_ij_* is the strain tensor, *ε_hy_* is the hydrostatic strain, *a_c_* is the conduction band hydrostatic deformation potential, *a_v_* is the valence band hydrostatic deformation potential, *b* is the deformation potential and *P*_0_ is the coupling between CB and VB. As discussed in Section 2.1, the average shear strain components were much smaller than the average hydrostatic strain, and all the off-diagonal elements were assumed to be zero.

For electrons, the strain induced potential is

*V_e_* = *a_c_ε_hy_* = *a_c_* (*ε_xx_* + *ε_yy_* + *ε_zz_*) (11)

For holes, we considered a 6 × 6 Luttinger–Kohn Hamiltonian matrix [18], and the strain-induced potentials for the heavy holes and light holes were [19]

*V_hh_* =−*P* − *Q* (12)

*V_lh_* = −*P* + [*Q* − Δ + (Δ^2^ + 2*Q*Δ + 9*Q*^2^)^½^]/2 (13)

where Δ is the spin-orbit splitting energy. Due to the lack of available data, *a_c_*, *a_v_* and m* of InPBi adopted the same value of InP, based on the consideration that the Bi content was small. The parameters used in the simulation are listed in Table 1.

## 4. Conclusions

In this paper, we proposed an InPBi QD/AlGaAs structure on a GaAs platform. It is predicted to be an effective way to increase the recombination efficiency and broaden the PL spectra of InPBi. FEM was used to calculate the strain distributions in the proposed structure. The hydrostatic strain was negative, and its absolute value increased with both height and Bi content. The band alignment was calculated based on the deformation potential theory. The CB, HHB and LHB of InPBi QD all rose under compressive strain. The energy differences between the CB and HHB of the InPBi QD increased with enhanced compressive strain. The energy of carrier recombination from electrons to the Bi-related deep level was enlarged. A high Bi content (x ≤ 2.7%) and a flat QD shape is preferable for broadening the PL spectra of InPBi, providing a feasible way to fabricate SLDs applied in OCT.

## Figures and Tables

**Table 1:** Parameters used for calculations.

Parameters	InP_1-*x*_Bi*_x_* [17]	Al_0.42_Ga_0.58_As [17]
C11 (GPa)	1011(1 − *x*) + 60.31*x*	1233.2
C12 (GPa)	561(1 − *x*) + 32.52*x*	552.56
C44 (GPa)	456(1 − *x*) + 26.1*x*	576.64
*a*_c_ (eV)	−6	−6.53
*a*_v_ (eV)	−0.6	−1.71
*b* (eV)	−2	−2.13
Δ(eV)	0.108 + 0.7*x* [5]	0.32
*m* $\overset{*}{e}$	0.0795 *m*_0_	0.098 *m*_0_ [20]
*m* $\overset{*}{\mathrm{hh}}$	0.6 *m*_0_	0.615 *m*_0_ [20]
*m* $\overset{*}{\mathrm{lh}}$	0.089 *m*_0_	0.11 *m*_0_ [20]

## References

[1] Alberi K., Wu J., Walukiewicz W., Yu K.M., Dubon O.D., Watkins S.P., Furdyna J. (2007). Valence-band anticrossing in mismatched III-V semiconductor alloys. Phys. Rev. B.

[2] Fluegel B., Francoeur S., Mascarenhas A., Tixier S., Young E.C., Tiedje T. (2006). Giant spin-orbit bowing in GaAs 1− x Bi x. Phys. Rev. Lett..

[3] Francoeur S., Adamcyk M., Tiedje T. (2003). Band gap of GaAs_1-x_Bi_x_, 0 < x < 3.6%. Appl. Phys. Lett..

[4] Wang K., Gu Y., Zhou H.F., Zhang L.Y., Kang C.Z., Wu M.J., Wang S.M. (2014). InPBi Single Crystals Grown by Molecular Beam Epitaxy. Sci. Rep..

[5] Kopaczek J., Kudrawiec R., Polak M.P., Scharoch P., Birkett M., Veal T.D., Wang S. (2014). Contactless electroreflectance and theoretical studies of band gap and spin-orbit splitting in InP1−xBix dilute bismide with x  ≤  0.034. Appl. Phys. Lett..

[6] Wu X., Chen X., Pan W., Wang P., Zhang L., Li Y., Wang S. (2016). Anomalous photoluminescence in InP1−xBix. Sci. Rep..

[7] Huang D., Swanson E.A., Lin C.P., Schuman J.S., Stinson W.G., Chang W., Puliafito C.A. (1991). Optical coherence tomography. Science.

[8] Zhang L., Wu M., Chen X., Wu X., Spiecker E., Song Y., Wang S. (2017). Nanoscale distribution of Bi atoms in InP 1−x Bi x. Sci. Rep..

[9] Zhang L., Song Y., Chen Q., Zhu Z., Wang S. (2018). InPBi Quantum Dots for Super-Luminescence Diodes. Nanomaterials.

[10] Pan W., Zhang L., Zhu L., Song Y., Li Y., Wang C., Wang S. (2016). Photoluminescence of InGaAs/GaAsBi/InGaAs type-II quantum well grown by gas source molecular beam epitaxy. Semicond. Sci. Technol..

[11] Pan W., Zhang L., Zhu L., Li Y., Chen X., Wu X., Wang S. (2016). Optical properties and band bending of InGaAs/GaAsBi/InGaAs type-II quantum well grown by gas source molecular beam epitaxy. J. Appl. Phys..

[12] Pan W., Wang P., Wu X., Wang K., Cui J., Yue L., Wang S. (2016). Growth and material properties of InPBi thin films using gas source molecular beam epitaxy. J. Alloys Compd..

[13] Rajpalke M.K., Linhart W.M., Birkett M., Yu K.M., Alaria J., Kopaczek J., Veal T.D. (2014). High Bi content GaSbBi alloys. J. Appl. Phys..

[14] Kuo C.P., Vong S.K., Cohen R.M., Stringfellow G.B. (1985). Effect of mismatch strain on band gap in III-V semiconductors. J. Appl. Phys..

[15] Chuang S.L. (2012). Physics of Photonic Devices.

[16] Kim K., Hart G.L.W., Zunger A. (2002). Negative band gap bowing in epitaxial InAs/GaAs alloys and predicted band offsets of the strained binaries and alloys on various substrates. Appl. Phys. Lett..

[17] Vurgaftman I., Meyer J., Ram-Mohan L. (2001). Band parameters for III–V compound semiconductors and their alloys. J. Appl. Phys..

[18] Sytnyk D., Melnik R. (2018). The Luttinger-Kohn theory for multiband Hamiltonians: A revision of ellipticity requirements. arXiv.

[19] Cicek B., Sánchez-Pérez J.R., Cavallo F., Lagally M.G., Paiella R. (2014). Strained-germanium nanostructures for infrared photonics. ACS Nano.

[20] Chen X., Wu X., Yue L., Zhu L., Pan W., Qi Z., Shao J. (2017). Negative thermal quenching of below-bandgap photoluminescence in InPBi. Appl. Phys. Lett..

